# A pragmatic lifestyle modification programme reduces the incidence of predictors of cardio-metabolic disease and dysglycaemia in a young healthy urban South Asian population: a randomised controlled trial

**DOI:** 10.1186/s12916-017-0905-6

**Published:** 2017-08-30

**Authors:** Mahen Wijesuriya, Nikolaos Fountoulakis, Nicola Guess, Sarath Banneheka, Laksha Vasantharajah, Martin Gulliford, Giancarlo Viberti, Luigi Gnudi, Janaka Karalliedde

**Affiliations:** 1 Diabetes Association of Sri Lanka, Colombo, Sri Lanka; 2 Cardiovascular Division, Faculty of Life Science & Medicine, King’s College London, London, UK; 3 Diabetes and Nutritional Sciences Division, Faculty of Life Science & Medicine, King’s College London, London, UK; 4 Department of Primary Care and Public Health Sciences, King’s College, London, UK

**Keywords:** Lifestyle modification, Diabetes prevention, South Asian, Cardio-metabolic disease, Younger participants

## Abstract

**Background:**

There is an increasing incidence of type 2 diabetes mellitus (T2DM) in young urban South-Asians. We tested the effect of a pragmatic trimonthly lifestyle modification (LSM) programme (P-LSM) versus a less-intensive 12-monthly control LSM (C-LSM) intervention on a primary composite endpoint of predictors of cardio-metabolic disease (new onset T2DM, hypertension, impaired glucose tolerance (IGT), impaired fasting glycaemia (IFG) and markers of cardio-renal disease) in participants aged 5–40 years with risk factors for T2DM.

**Methods:**

This was a randomised controlled trial performed at the National Diabetes Centre, Sri-Lanka. We individually randomised 4672 participants at risk of T2DM, of whom 3539 (mean age 22.5 (range 6–40 years, 48% males) received either trimonthly (P-LSM *n* = 1726) or 12-monthly (C-LSM *n* = 1813) peer educator advice aimed at reducing weight, improving diet, reducing psychological stress and increasing physical activity.

**Results:**

During a median follow-up of 3 years, the cumulative incidence of the primary endpoint was *n* = 479 in P-LSM (74 per 1000 person years) vs. 561 in C-LSM (96 per 1000 person years), with an incident rate ratio (IRR) of 0.89 (95% CI 0.83–0.96, *P* = 0.02). In post hoc analyses, new onset dysglycaemia (T2DM, IFG and IGT), was the major contributor to the composite and was significantly reduced by P-LSM (IRR 0.9, 95% CI 0.83–0.97, *P* = 0.01). A significant impact of P-LSM on the incidence of the composite endpoint was noted in 1725 participants (P-LSM *n* = 850, C-LSM *n* = 875) aged below 18; P-LSM *n* = 140 (48 per 1000 person years) versus C-LSM *n* = 174 (55.4 per 1000 person years), with an IRR of 0.83 (95% CI 0.73–0.94, *P* = 0.004).

**Conclusions:**

In a young at-risk South-Asian population, a pragmatic LSM programme significantly reduces the incidence of predictors of cardio-metabolic disease. Our results highlight the importance of early intervention in young at-risk subjects.

**Trial registration:**

World Health Organization international clinical trial registry platform (SLCTR/2008/003). Registration Date: March 28, 2008. Retrospectively registered.

**Electronic supplementary material:**

The online version of this article (doi:10.1186/s12916-017-0905-6) contains supplementary material, which is available to authorized users.

## Background

The number of people with type 2 diabetes mellitus (T2DM) is expected to reach 592 million by the year 2035, with 80% of all persons with diabetes living in low- to middle-income countries [1, 2]. In parallel, the number of adults with impaired glucose tolerance (IGT) will rise from 316 million in 2013 to an estimated 471 million by 2035 [1]. Asia is the major site of the epidemic of T2DM, accounting for 60% of the people with diabetes worldwide [1]. South Asians are predisposed to early onset of T2DM, with almost a third of future T2DM cases predicted to occur in those aged below 45 years [2].

Sri Lanka is a middle-income Asian country with a population of 20.3 million. Approximately 40% and 25% of Sri Lankans are aged below 40 and 18 years, respectively [3]. The prevalence of T2DM in urban Sri Lankans is of 16.4% [4]. Lifestyle modification (LSM) has been demonstrated to prevent the onset of T2DM in older participants (aged > 40 years) with IGT [5–8]. Nevertheless, the effect of LSM on the onset of cardio-metabolic disease in younger healthy participants has not been studied to date.

## Methods

### Setting and participants

Prevention of cardio-metabolic disease with lifestyle modification in Sri Lanka (DIABRISK-SL) was a randomised controlled clinical trial comparing a trimonthly pragmatic LSM (P-LSM) programme with a less-intensive 12 monthly LSM (C-LSM) programme on a primary composite endpoint of predictors of cardio-metabolic disease in young urban healthy participants selected using a ‘high-risk’ screening strategy.

We have previously reported in detail the design of the study, trial protocol and baseline characteristics of the cohort studied [9, 10]. Participants were recruited following screening of a population of 23,298 representative of the general population of Colombo District, the most populous urban district in Sri Lanka.

The screening strategy has been described in detail previously [9]. The screening survey took place between January 1, 2008, and June 30, 2009. More than 95% of subjects and institutions approached agreed to participate.

Participants aged between 5 and 40 years were screened to identify the prevalence of four risk factors, namely first degree (parental) family history of T2DM, physical inactivity, raised body mass index (BMI), and raised waist circumference (WC). In participants aged between 5 and 17 years, raised WC was defined as ≥ 91st percentile for sex and age, whereas in subjects aged 18–40 years it was defined as ≥ 80 cm in females and as ≥ 90 cm in males. Raised BMI was defined as a value greater than internationally standardised age- and sex-specific percentile cut-offs in participants aged 5–17 years and as BMI ≥ 23 kg/m^2^ in those aged between 18 and 40 years [9]. Physical activity was measured using physical activity questionnaire (IPAQ short version) and inactivity defined as less than 30 minutes continuous exercise for less than 3 days a week [9]. In participants younger than 15 years of age, where IPAQ is not fully validated, the parents or guardians corroborated what was reported.

In the screening phase results previously reported [10], the prevalence of physical inactivity was nearly 52% in those aged > 20 years. Participants with two or more risk factors identified in the screening phase were invited to take part in the clinical trial.

The inclusion criterion for the trial was participants with two or more of the above risk factors. Exclusion criteria were participants with no or one identifiable risk factor, participants with diagnosed end-points (T2DM, cardiovascular disease (CVD), hypertension and renal disease), participants on any form of medication used for the treatment of diabetes, hypertension, renal disease or dyslipidaemia, active communicable or non-communicable diseases such as cancer, asthma or other forms of chronic lung disease, depression, tuberculosis and pregnancy.

All participants provided written informed consent and the study was given ethical approval from the Sri Lanka Medical Association Ethical Review Committee (ERC 07-010). Permission from the Ministry of Education was obtained for this study, which was conducted under the Good Clinical Practice Guidelines and according to the principles expressed in the Declaration of Helsinki for clinical research.

### Randomisation and interventions

Following screening of 23,298 participants, 5156 were identified as being eligible for the trial. Of the 5156 participants invited to take part in the trial, 4672 participants attended the National Diabetes Centre where standardised anthropometric and biochemical testing were performed as previously described [9]. No lifestyle program/interventions were delivered at this visit and participants were randomised into two intervention trial arms P-LSM or C-LSM using a computer generated randomisation schedule/sequence (SPSS Inc., 233 South Wacker Drive, 11th floor, Chicago, IL 60606-6412). There were no restrictions such as blocking and block size. Allocation into the two trial arms were implemented using sealed envelopes. Enrolment and assignment of participants was performed by a member of the research team. Participants and researchers were not blinded to group assignment. The CONSORT diagram showing the flow of participants is shown in Additional file 1: Figure S1. The median (range) follow-up was 3 (1-4) years.

### Intervention and goals

All participants received an identical lifestyle education programme, with the only difference being the frequency of the delivery of this advice (3 monthly or 12 monthly). In the P-LSM group, advice was given every 3 months, on a one-to-one basis, on the importance of a healthy diet and lifestyle with the aim of reducing weight, encouraging regular exercise and managing psychological stress, as described in detail previously [9]. Briefly, participants with a raised BMI or WC were set a target of > 5% weight loss over 12 months. In children, the aim was to limit excessive weight gain. Participants were advised to avoid a high intake of foods containing simple sugars, refined carbohydrates and high saturated fats (total fat < 20 g/day) and recommended to increase their intake of fibre-rich natural foods such as wholegrains, legumes, vegetables and fruits.

Participants with physical inactivity at baseline were advised to increase physical activity to at least 30 minutes of continuous exercise a day for more than 5 days a week [11]. Children were advised on the need to play sports they enjoyed (examples included running, netball, cricket, badminton) for at least 1 hour every day [9, 11]. Sedentary activities such as playing computer games and watching television were discouraged in all participants.

Peer educators were selected following local advertisements for the positions, and were trained by specialist nutritionists and diabetes specialists for 4 weeks prior to starting work. The selection criteria were young persons (aged 18 to 40 years) living in Colombo District with secondary school education (with three advanced level passes in studied subjects), and good communication skills. A training manual was developed for the peer educators and their training supervised by University of Colombo staff, and two external experts on lifestyle modification from South India. The standardised training manual was used by all the peer educators as a guide to deliver the interventions.

Criteria for failing training included an inability to demonstrate a sound understanding of the lifestyle programme and intervention, and poor communication skills after the training period. Approximately 20 peer educators were trained during the study with a drop-out rate of 25%. Monthly training refresher sessions were performed throughout the study with peer-to-peer education actively encouraged.

With regards to receipt of intervention, this component of fidelity was evaluated by recording attendance at sessions, adherence to goals set and impact of the intervention on motivation by assessing behaviour change and readiness to change as detailed below. Each session lasted at least 30 minutes. In younger children (age < 16 years) the LSM advice and guidance was also given to the child’s parents. A readiness to change ruler and questionnaire was utilised to assess behaviour change [12]. Participants were asked about their readiness to change before assessing the results. These parameters were used to facilitate the participants’ understanding of the importance of behaviour change and to aid the delivery of the intervention. The stages of behaviour change were defined as pre-action (pre-contemplation, contemplation and preparation) and action (action and maintenance), using the trans-theoretical model of behaviour change [12].

The presence or absence of stress and depression was assessed at each visit using the Perceived Stress Score Questionnaire and the Patient Health Questionnaire 9 [10]. Participants were provided counselling and advice on pragmatic strategies to reduce stress. It was emphasised that improving lifestyle would have a positive impact on perceived stress and anxiety levels over the duration of the study.

All participants in the intensive group received trimonthly individualised LSM advice to assess progress (P-LSM). If needed, goals were reiterated and reinforced. The control group (C-LSM) received lifestyle advice annually, which was identical in format and structure to that provided to participants in the P-LSM group. The control group received annual LSM counselling as it would have been unethical to withhold any intervention in participants considered to be at future risk.

### Outcomes and follow-up

The primary composite cardio-metabolic endpoint was new onset T2DM, hypertension, IGT, impaired fasting glycaemia (IFG), CVD and renal disease. New cases of T2DM (fasting plasma glucose ≥ 126 mg/dL (7.0 mmol/L) or an oral glucose tolerance test (OGTT) with the 2-h post-load value ≥ 200 mg/dL (11.1 mmol/L)), IGT (2-h post load plasma glucose ≥ 140 (7.8 mmol/L) and < 200 mg/dL (11.1 mmol/L)), IFG (fasting plasma glucose ≥ 100  (5.6 mmol/L) and < 126 mg/dL (7.0 mmol/L)) were diagnosed according to the American Diabetes Association criteria [11, 13].

Endpoints were diagnosed at the time of follow-up visits or if made by the participant’s family physician and confirmed on repeat testing. Participants with IFG or IGT diagnosed at baseline evaluation were eligible to participate in the trial. In these subjects, development of T2DM was the metabolic endpoint. Participants with endpoint diagnoses, including diagnosis of diabetes, underwent medical and clinical review. All participants with a new diagnosis of diabetes had a clinical history and features of T2DM. No participant with a diagnosis of new onset diabetes had clinical or biochemical features indicative of type 1 diabetes. Furthermore, none of the participants diagnosed required insulin treatment or displayed any features of insulin deficiency.

In adults, new cases of hypertension were defined as brachial blood pressure ≥ 140/90 mmHg; in subjects aged below 18 years, hypertension was defined following Joint National Committee 7 guidelines (blood pressure that is, on repeated measurement, at the 95th percentile or greater adjusted for age, height and sex) [12]. Renal disease was defined as new onset microalbuminuria (urine albumin/creatinine ratio of ≥ 22 mg/g in men and ≥ 31 mg/g in women confirmed on one repeat test) or new onset renal impairment defined as estimated GFR < 60 mL/min [9]. Initiation of statin therapy was included in the primary composite endpoint in view of recent substantive data that this class of drugs can influence glucose levels [14]. Because of the young age range of our study population the composite outcome was composed of predictors/risk factors for clinical events with the addition of CVD events and all cause death. Participants were asked about events and only positive events were followed by review of hospital records. CVD event information was obtained from local hospital records, and when participants gave a positive response they were asked about events.

The choice of risk factors in the primary outcome is clearly relevant to the younger age groups as occurrence of these risk factors has been shown to occur in childhood and track with time [15, 16].

### Statistical analysis

No information was available in a population comparable to the one reported on in this proof of concept work and hence the power calculation was largely hypothesis driven and based on risk reductions for new onset T2DM in older IGT subjects observed with more intensive LSM from South Asia [7]. We assumed a 15% incidence of the primary end-point in the C-LSM group and estimated that approximately 2300 participants in each group will enable the detection of at least 25% relative risk reduction in the primary endpoint with P-LSM, at the 90% power level with a type-1 error of 0.05 (two-sided) [9]. Descriptive statistics were used for the analysis of demographic and clinical features of the cohort. Comparison of proportions was performed by χ^2^ analysis. Analyses of the incidence of dysglycaemia and the impact of the intervention in participants above and below 18 years of age were not pre-specified and performed post hoc. Regression analyses were used to estimate mean differences between the groups at the end of the study, which were adjusted for baseline value. For the biomarker endpoints, the last observation was carried forward for those participants with missing endpoint values who did not complete the full duration of the trial. There was no interim analysis performed.

The final analysis was in the intent-to-treat population, who were randomised and had at least one post-randomisation visit/evaluation where intervention was delivered. We also report analyses for all participants who attended randomisation irrespective of whether they attended their first post-randomisation visit.

We had originally proposed to perform survival analysis to estimate the cumulative incidence end-points and test the difference between the groups by means of the two-sided log-rank test. However, we updated the analyses plan to Poisson multivariate regression analyses (analyses were performed by the statistician on blinded data set) rather than survival analyses due to the following reasons. The Poisson approach is suitable when analysing time to event data when times between successive events are independent and exponentially distributed and the distribution of follow-up times similar for both groups (as was the case in our data set). In this scenario, a survival analysis would not be the most robust form of analyses because end points could not be observed continuously. Moreover, the Poisson method enables an efficient method to deal with cumulative exposure and other time-dependent covariates and allows for risk to depend on multiple timelines [17, 18]. Poisson regression analyses were performed, with person-time as exposure, to estimate the incident rate ratio (IRR) with P-LSM as compared to C-LSM. Analyses were performed using SPSS software version Version 20.0. Armonk, NY, USA. Data are given as mean with 95% confidence intervals (CI) unless otherwise stated. A *P* value of less 0.05 was considered significant.

## Results

The clinical and biochemical features of the screened population have been previously reported [10]. In brief, of the 23,298 participants screened, 5156 were eligible and 4672 participants attended the National Diabetes Centre to have baseline assessments and received the allocated intervention.

Of the 4672 participants, 3539 (76%) were eligible for analysis. In total, 1133 participants (approximately 24% of subjects, similarly distributed between trial arms) were excluded from the analysis as they failed to attend any LSM advice visits after the baseline randomisation visit. These participants had a similar mean age and prevalence of baseline risk factors as compared to participants who attended visits post randomisation, but had modestly higher blood pressure levels and lower glucose levels (Additional file 2: Table S1).

The baseline clinical and biochemical characteristics of participants in the two trial arms were similar, with no statistically significant differences between the two arms (Table 1). The prevalence of raised BMI, raised WC, and parental family history of T2DM in the P-LSM and in the C-LSM trial arms were similar at baseline. The prevalence of physical inactivity strictly by definition was 100% in both arms (as all participants eligible to enter clinical trial reported less than 30 minutes continuous exercise for less than 3 days a week). The number of participants who were tobacco smokers and consumed alcohol were comparable in the two trial arms (Table 1).

**Table 1:** Baseline characteristics of 3539 healthy participants who received pragmatic lifestyle modification or control lifestyle modification

Characteristics	Pragmatic lifestyle modification	Control lifestyle modification
Number	1726	1813
Age, mean (range) years	22.5 (6–40)	22.4 (7–40)
Sex, %		
Males	837 (48.5%)	877 (48.4%)
Female	889 (51.5%	936 (51.6%)
Prevalence of raised BMI, %	69.1	69.8
Prevalence of raised WC, %	63.0	64.4
Prevalence of a FH of T2DM, %	52.0	52.1
Prevalence of physical inactivity, %	100	100
Impaired fasting glycaemia, %	7.8	8.2
Impaired glucose tolerance, %	7.9	8.3
Smokers	5.7%	6.4%
Alcohol use	16%	17%
Waist circumference, cm	84.3 (83.7–84.8)	84.1 (83.5–84.5)
Body mass index, kg/m^2^	24.0 (23.8–24.2)	24.0 (23.7–24.1)
Systolic blood pressure, mmHg	114.1 (113.8–115.1)	114.0 (113.8–115.0)
Diastolic blood pressure, mmHg	71.0 (70.5–71.5)	71.1 (70.9–71.9)
Fasting plasma glucose, mmol/L	4.95 (4.9–5.0)	4.95 (4.9–5.0)
2-hour post-oral glucose tolerance test plasma glucose, mmol/L	5.84 (5.8–5.9)	5.91 (5.8–6.0)
Total cholesterol, mmol/L	4.95 (4.90–4.96)	4.96 (4.92–5.0)
Low density lipoprotein, mmol/L	3.19 (3.15–3.22)	3.22 (3.19–3.26)
High density lipoprotein, mmol/L	1.14 (1.13–1.15)	1.14 (1.13–1.16)
Serum triglycerides, mmol/L	1.25 (1.22–1.28)	1.27 (1.24–1.31)

Data are mean and 95% confidence intervals unless otherwise stated

There were no statistically significant differences between the two trial arms

*BMI* body mass index, *WC* waist circumference, *FH* family history, *T2DM* type 2 diabetes mellitus

At baseline, there was a similar prevalence of IFG and IGT. Stage of behaviour change for diet, exercise and stress were similar at baseline in both groups and more than 80% of participants were in the pre-action phase.

Table 2 details the number of participants in the P-LSM and C-LSM groups who developed the respective individual components of the primary composite endpoint in the whole cohort with the respective IRR comparing the two groups. After a median follow-up of 3 years, the cumulative incidence of the primary composite cardio-metabolic endpoint was *n* = 479 in P-LSM (74 per 1000 person years) versus 561 in C-LSM (96 per 1000 person years), with an IRR of 0.89 (95% CI 0.83–0.96, *P* = 0.002) (Table 2).

**Table 2:** Effect of pragmatic lifestyle modification as compared to control lifestyle modification on the incidence of the primary cardio-metabolic composite endpoint and its individual components in 3539 healthy participants

Component of primary composite end-point	Pragmatic lifestyle modification *n* = 1726	Control lifestyle modification *n* = 1813	Incident rate ratio (95% confidence intervals)	*P* value
Composite	479	561	0.89 (0.83–0.96)	0.002
New onset T2DM	58	72	0.8 (0.65–1.02)	0.08
New onset IGT	143	168	0.89 (0.79–1.01)	0.08
New onset IFG	146	166	0.93 (0.82–1.06)	0.27
New onset dysglycaemia^a^	347	406	0.9 (0.83–0.97)	0.013
Hypertension	115	152	0.79 (0.68–0.9)	0.01
Statin therapy	41	46	0.92 (0.72–1.18)	0.5
Renal disease events	2	8	0.26 (0.1–0.63)	0.003
Cardiovascular events	1	0		N/A
Deaths	2	0		N/A

^a^Composite of T2DM, IFG and IGT. *T2DM* type 2 diabetes mellitus, *IGT* impaired glucose tolerance, *IFG* impaired fasting glycaemia, *N/A* not applicable

New onset hypertension was significantly reduced with P-LSM (IRR 0.79, 95% CI 0.68–0.9, *P* = 0.01). In comparison with the C-LSM, fewer patients developed new onset T2DM, IFG and IGT with P-LSM; however, the risk reductions observed were not significant.

New onset dysglycaemia (defined post hoc as a composite of T2DM, IFG and IGT) was the major contributor to the composite and was significantly reduced by P-LSM (IRR 0.9, 95% CI 0.83–0.97, *P* = 0.01).

Table 3 shows the post hoc analyses results and breakdown of the individual components of the primary composite endpoint in those above and below 18 years of age with the respective IRR comparing the two groups.

**Table 3:** Effect of pragmatic lifestyle modification as compared to control lifestyle modification on the incidence of the primary cardio-metabolic composite endpoint and its individual components in 1814 healthy participants aged above 18 and 1725 below 18 years of age

Component of primary composite endpoint	Pragmatic lifestyle modification	Control lifestyle modification	Incident rate ratio (95% confidence Intervals)	*P* value
Participants aged ≥ 18 years	*n* = 876	*n* = 938		
Composite	339	387	0.93 (0.86–1.03)	0.11
New onset T2DM	54	63	0.9 (0.73–1.11)	0.35
New onset IGT	97	105	1.01 (0.87–1.17)	0.89
New onset IFG	88	115	0.78 (0.67–0.91)	0.02
New onset dysglycaemia^a^	239	283	0.9 (0.82–1.00)	0.051
Hypertension	82	95	0.91 (0.77–1.089)	0.31
Statin therapy	38	45	0.89 (0.69–1.15)	0.39
Renal disease events	0	4		N/A
Cardiovascular events	1	0		N/A
Deaths	1	0		N/A
Participants aged < 18 years	*n* = 850	*n* = 875		
Composite	140	174	0.83 (0.73–0.94)	0.004
New onset T2DM	4	9	0.48 (0.24–0.94)	0.032
New onset IGT	46	63	0.74 (0.60–0.90)	0.009
New onset IFG	58	51	1.13 (0.93–1.4)	0.13
New onset dysglycaemia^a^	113	133	0.91 (0.79–1.03)	0.24
Hypertension	33	57	0.60 (0.47–0.76)	0.001
Statin therapy	3	1	2.89 (0.77–10.7	0.11
Renal disease events	2	4	0.5 (0.19–1.33)	0.16
Cardiovascular events	0	0		N/A
Deaths	1	0		N/A

^a^Composite of T2DM, IFG and IGT. *T2DM* type 2 diabetes mellitus, *IGT* impaired glucose tolerance, *IFG* impaired fasting glycaemia, *N/A* not applicable

The impact of P-LSM on the incidence of the composite endpoint was observed in participants aged below 18; P-LSM *n* = 140 (48 per 1000 person years) versus C-LSM *n* = 174 (55.4 per 1000 person years), with an IRR of 0.83 (95% CI 0.73–0.94, *P* = 0.004). A significant reduction in new onset IGT (P-LSM *n* = 46 vs. C-LSM *n* = 63, IRR 0.74, 95% CI 0.6–0.9, *P* = 0.009), T2DM (P-LSM *n* = 4 vs. C-LSM *n* = 9, IRR 0.48, 95% CI 0.24–0.94, *P* = 0.03) and hypertension (P-LSM *n* = 33 vs. C-LSM *n* = 57, IRR 0.6, 95% CI 0.47–0.76, *P* = 0.001) was also observed with P-LSM in participants aged below 18 years of age (Table 3).

In those participants above 18 years of age, a significant reduction in new onset IFG was observed with P-LSM (*n* = 88) versus C-LSM (*n* = 115), with an IRR of 0.78 (95% CI 0.67–0.91, *P* = 0.02), and a trend toward less new onset dysglycaemia P-LSM (*n* = 253) versus C-LSM (*n* = 303), with an IRR of 0.9 (95% CI, 0.82 to 1.00, *P* = 0.051).

In post-hoc analyses of all participants who were randomised (including those who failed to attend for any LSM advice after the baseline randomisation visit), the results for the primary endpoint demonstrated a similar significant risk reduction with P-LSM (IRR 0.86, 95% CI 0.80–0.93, *P* = 0.001) and risk reductions comparable to what we detail above were also observed for the individual  components of the primary endpoint.

End of study values for selected clinical and biochemical variables, adjusted for baseline value, age and sex for participants above and below 18 years of age in the P-LSM and C-LSM groups are shown in Additional file 2: Table S2. At the end of the trial, there was a small but statistically significant lower fasting plasma glucose and 2-h post-OGTT plasma glucose level in the P-LSM as compared to the C-LSM group in participants aged above 18 years of age only (*P* < 0.05 for both). Blood pressure, anthropometric and lipid parameters were not significantly different between the two groups at the end of the study.

The percentage of participants adhering to LSM advice and achieving weight loss and improving related behaviour change from baseline was similar in both groups (Additional file 2: Table S3). Physical activity (defined as > 600 MET/min-week) increased from a baseline of 39% in both groups to 47.2% in P-LSM and 42% C-LSM, with a significant difference between groups in participants both above and below 18 years of age. There was also a significant effect of P-LSM as compared to C-LSM to increase behaviour change with regards to increasing physical activity, an effect that was observed in participants above and below 18 years of age (Additional file 2: Table S3).

### Adverse events

There were two deaths during the study, both in P-LSM (the causes of death were road traffic accident and haematological malignancy), and one CVD event in P-LSM.

## Discussion

The results from our translational proof of concept trial demonstrates that a peer educator-delivered P-LSM intervention of trimonthly advice results in a significantly lower incidence rate of predictors of cardio-metabolic endpoints in young South Asians with risk factors for T2DM and CVD. The majority of endpoint events were metabolic glucose abnormalities, with a significant reduction in new onset dysglycaemia (new onset T2DM, IFG and IGT) with P-LSM. We also observed significant reduction in new onset hypertension with P-LSM. Interestingly, the beneficial effects of P-LSM on the primary composite cardio-metabolic endpoint, new onset T2DM, new IGT and hypertension were observed in participants below 18 years of age.

The cumulative incidence of T2DM we observed was much lower than that observed in previous studies of LSM such as the Diabetes Prevention Program (DPP) [5] in the United States, Finnish Diabetes Prevention study (DPS) [6], the Indian Diabetes Prevention Programme (IDPP) [7], and the Da Qing Diabetes Prevention study [8]. These seminal studies focussed on higher risk, older (mean age 50 years) participants with baseline IGT [5–8]. In contrast, our study was focused on younger (mean age 22.5 years), healthier participants, of whom 92% had normal glucose tolerance at baseline.

The control group in our study received goal-orientated LSM advice with the same targets as the P-LSM group, albeit at a lower intensity. This would have contributed to the smaller relative risk reductions and incident rates of T2DM we observed as compared to previous studies, and could result in an underestimation of the true risk reduction with the P-LSM intervention [5].

It is reasonable to suggest that reduction in risk with P-LSM of dysglycaemia and hypertension, two conditions causally related to CVD, may translate over time into fewer cardiovascular events [19, 20]. However, this remains to be demonstrated in younger populations such as ours, but recent data from the longer term follow-up of the Da Qing study cohort supports this premise [21].

The main strengths of our study are the young healthy population, a sample not previously evaluated in the context of diabetes prevention, the large number of participants and the relatively long follow-up period. Our study is the first demonstration in participants below the age of 18 years that lifestyle modification delivered by peer educators reduces the development of predictors of cardio-metabolic disease. We also used a novel delivery of LSM advice on a one-to-one basis by peer educators, which was very effective.

The cost of peer educators to deliver the intervention would be significantly lower than recruiting registered dietitians or behaviour-change counsellors as has been used in other trials [5]. We contend that such a pragmatic, cost-effective approach is therefore translatable to other developing and middle-income countries. We can also speculate that this younger population may be more ‘responsive’ and amenable to change compared to older participants, particularly when delivered by peers. Importantly, any reduction in the future risk of dysglycaemia and hypertension would be of significant health impact, especially in view of the growing worldwide burden of cardio-metabolic disease in younger populations [2, 10].

There are limitations to our study. The specific mechanisms that explain the reduction in the primary cardio-metabolic endpoint with the P-LSM intervention are unclear. In our trial, P-LSM had no effect on weight loss. The Da Qing study demonstrated no significant weight loss and that weight change was not related to reduction in new onset T2DM [8]. Similar results were observed in the IDDP [7]. These results from Asian participants are at variance with results from predominantly Caucasian participants studied in the North American DPP and Finnish DPS, in which the impact of lifestyle modification on preventing T2DM was mostly driven by weight loss, though physical activity also increased [5, 6, 22].

Our P-LSM intervention resulted in increased physical activity and improved behaviour change to increase physical activity and this was true for participants both above and below 18 years of age. Exercise, even in the absence of weight loss, is known to improve insulin sensitivity, which is a key determinant of the 2-hour glucose concentration following an OGTT [23, 24]. However, our study was not designed to examine the mechanism of effect or test the relative contributions of weight change and physical activity on cardio-metabolic endpoints. Our results indicate that, at least in young healthy South Asian participants, weight loss per se is not essential for reducing the risk of cardio-metabolic disease and we can speculate that increased physical activity may be an important factor [2, 7, 22]. Our findings reinforce the need for future research in this area.

There was little evidence of improvement in metabolic variables with P-LSM. These results reflect the relatively healthy baseline population we studied as compared to other T2DM prevention trials [25–27].

Our findings in a young urban population may not be generalisable to other groups. The annual structured LSM advice received by the C-LSM control group may have resulted in an underestimation of the risk reduction that would be seen with P-LSM under ordinary circumstances. An alternative view could be that modest risk reduction observed may be realistic in view of public information on healthy living currently available in urban Sri Lanka.

Attrition could have caused bias if those with symptoms were more likely to return for follow-up. However, participants might have also defaulted because they were too ill to attend. We have no evidence for or against either of these situations. Nearly 24% of randomised participants were not eligible for analyses as they did not attend any visits to receive lifestyle advice and we had no follow-up data available on them. These participants had similar baseline features as compared to those who were eligible for analyses but the lack of follow-up information is a significant limitation of our work. The analyses of participants below 18 years was not pre-specified; however, our results establish the platform and rationale for further studies in this population.

We chose trimonthly visits in the P-LSM trial arm as this was the most feasible and pragmatic intervention schedule/programme that could be delivered in the context of the study population, with the available resources. Previous studies, such as the DPP, DPS and IDPP were proven to be cost effective. Our relatively conservative P-LSM programme with four visits a year undertaken by young peer educators is likely to be at least as cost-effective, but detailed cost-benefit and economic analyses are required and planned.

## Conclusions

We take the view that, in South Asian subjects, who are known to be at enhanced and premature risk of T2DM and CVD, early interventions that can delay or prevent onset of cardio-metabolic endpoints are of clinical importance. Longer term follow-up of the DPP and Da Qing study cohorts showed a lasting effect of the original life change intervention with a significant reduction in new onset T2DM up to 10–14 years later [28, 29] and cardiovascular and all-cause mortality [21]. This suggests a significant legacy effect of the intervention and emphasises the importance of early intervention especially in a younger at-risk group. Recent publications highlight the urgent need and beneficial impact of diabetes prevention and implementation into general practice and promote the concept that even small changes in lifestyle can result in big changes in health [30, 31].

In summary, this is the first study to prove that in an at-risk urban South Asian population, across a wide range of young ages, a pragmatic lifestyle modification programme can significantly reduce the development of risk factors that are causally related to future development of T2DM and CVD in later life. Our study highlights the importance of early intervention and establishes the proof of concept and rationale for further pragmatic lifestyle modification intervention studies in young South Asian participants at risk of cardio-metabolic disease.

## Additional files

Additional file 1: Figure S1. CONSORT diagram showing the flow of participants through each stage of the DIABRISK-SL randomised controlled trial. (DOCX 26 kb)

Additional file 2: Table S1. Comparison of baseline characteristics of 3539 healthy participants who were eligible for analyses with 1133 participants who were ineligible for analyses as they as did not attend post-randomisation visits. **Table S2.** End-of-study metabolic and haemodynamic parameters and mean adjusted difference (pragmatic lifestyle modification (P-LSM) as compared to control lifestyle modification (C-LSM)) in 1814 healthy participants aged above 18 years and 1725 below 18 years of age. **Table S3.** Percentage of participants adhering to and achieving weight loss, physical activity goals and behaviour change for physical activity and stress reduction: P-LSM as compared to C-LSM in 3539 healthy participants. (DOC 100 kb)
