# Supplementary material for: A pragmatic lifestyle modification programme reduces the incidence of predictors of cardio-metabolic disease and dysglycaemia in a young healthy urban South Asian population: a randomised controlled trial
Source: BMC Med. 2017 Aug 30;15:146. doi: 10.1186/s12916-017-0905-6 (PMC5576225; doi:10.1186/s12916-017-0905-6)
Supplement: Supplementary file 1 — CONSORT diagram showing the flow of participants through each stage of the DIABRISK-SL randomised controlled trial. (DOCX 26 kb) [file 12916_2017_905_MOESM1_ESM.docx]

Figure S1: CONSORT diagram showing the flow of participants through each stage of the Diabrisk-SL randomized controlled trial.

Assessed for eligibility n=23298

Excluded as not meeting inclusion criteria (n =18142)

Eligible for trial n=5156

Allocated Pragmatic life style modification intervention n=2346

**Allocation**

Allocated Control life style modification intervention n=2326

**Follow up**

Lost to follow up (n =387)

Pregnancy (n=129)

Migration (n=104)

Lost to follow up (n=314) Pregnancy (n=130)

Migration (n=69)

Analysed

n=1726

Analysed

n=1813

**Analysed**

**Enrolment** Allocation

Refused to participate in trial (n =484)
