# Supplementary material for: A pragmatic lifestyle modification programme reduces the incidence of predictors of cardio-metabolic disease and dysglycaemia in a young healthy urban South Asian population: a randomised controlled trial
Source: BMC Med. 2017 Aug 30;15:146. doi: 10.1186/s12916-017-0905-6 (PMC5576225; doi:10.1186/s12916-017-0905-6)
Supplement: Supplementary file 2 — Comparison of baseline characteristics of 3539 healthy participants who were eligible for analyses with 1133 participants who were ineligible for analyses as they as did not attend post-randomisation visits. Table S2. End-of-study metabolic and haemodynamic parameters and mean adjusted difference (pragmatic lifestyle modification (P-LSM) as compared to control lifestyle modification (C-LSM)) in 1814 healthy participants aged above 18 years and 1725 below 18 years of age. Table S3. Percentage of participants adhering to and achieving weight loss, physical activity goals and behaviour change for physical activity and stress reduction: P-LSM as compared to C-LSM in 3539 healthy participants. (DOC 100 kb) [file 12916_2017_905_MOESM2_ESM.doc]

Table S1 Comparison of baseline characteristics; 3539 healthy participants who were eligible for analyses with 1133 who were ineligible for analyses as they as did not attend post randomisation visit.

| Characteristics | Eligible for analyses  N = 3539 | Ineligible for analyses N=1133 | p-value |
| --- | --- | --- | --- |
| Age mean years | 22.1± 10.0 | 22.2±9.2 | 0.90 |
| Raised body mass index (%) | 2551 (70%) | 603 (70%) | 0.80 |
| Raised waist circumference (%) | 2354 (64%) | 556 (64%) | 0.84 |
| Physical inactivity (%) | 3685 (100%) | 867 (100%) | 0.23 |
| First degree family history (%) | 1902 (52%) | 463 (53.4%) | 0.31 |
| Waist circumference, cm | 84.3 ±11.3 | 84.6 ±11.32 | 0.32 |
| Body mass index, kg/m2 | 24.0 ±4.4 | 24.2 ±4.5 | 0.20 |
| Systolic blood pressure, mmHg | 114.1 ± 12.8 | 116.4±14.7 | 0.001 |
| Diastolic blood pressure, mmHg | 71.0 ± 10.3 | 72.3±11.2 | 0.003 |
| Fasting plasma glucose, mmol/l | 4.95± 0.4 | 4.89±0.5 | 0.01 |
| 2 hour post oral glucose tolerance test plasma glucose, mmol/l | 5.83 ± 1.3 | 5.78± 1.3 | 0.08 |

Data are mean ± SD unless otherwise stated

Table S2: End-of-study metabolic and haemodynamic parameters and mean adjusted difference (Pragmatic lifestyle modification as compared to control lifestyle modification) in 1814 healthy participants aged above 18 years and 1725 below 18 years of age

| **Parameter**  **at end of study** | **Pragmatic**  **lifestyle modification** | **Control**  **lifestyle modification** | **Adjusted Difference**  **(95% CI)** | **P value** |
| --- | --- | --- | --- | --- |
| **Participants aged ≥ 18 years** | n=876 | n=938 |  |  |
| Weight  kg | 69.7±13.4 | 70.04±12.8 | 0.13 (-0.25 to 0.52) | 0.48 |
| Waist circumference cm | 91.0±9.6 | 91.6±9.3 | 0.25 (-0.18 to 0.68) | 0.26 |
| Body mass index kg/m^2^ | 25.8±4.0 | 25.9±3.7 | 0.04 (-0.09 to 0.18) | 0.54 |
| SBP mmHg | 113.1±12.2 | 113.7±11.9 | 0.73 (-0.22 to 1.69) | 0.13 |
| DBP mmHg | 71.2±9.1 | 71.7±9.2 | 0.35 (-0.39 to 1.11) | 0.30 |
| Fasting plasma glucose  mmol/l | 5.10±0.7 | 5.13±1.0 | 0.07 (0.004 to 0.144) | 0.04 |
| 2 hour post oral glucose tolerance test plasma glucose  mmol/l | 6.41±2.2 | 6.63±2.6 | 0.21 (0.007 to 0.406) | 0.04 |
| Total cholesterol  mmol/l | 5.41±0.94 | 5.47±0.95 | -0.001 (-0.06 to 0.06) | 0.9 |
| LDL mmol/l | 3.56±0.85 | 3.63±1.4 | 0.03 (-0.06 to 0.12) | 0.55 |
| HDL mmol/l | 1.19±0.26 | 1.19±0.26 | -0.007(-0.021to 0.008) | 0.38 |
| Serum triglycerides mmol/l | 1.42±0.7 | 1.49±0.97 | 0.05 (-0.009 to 0.109) | 0.09 |
|  |  |  |  |  |
| **Parameter**  **at end of study** | **Pragmatic**  **lifestyle modification** | **Control**  **lifestyle modification** | **Adjusted Difference**  **(95% CI)** | **P value** |
| **Participants aged < 18 years** | n=850 | n=875 |  |  |
| Weight  kg | 65±15.6 | 65.7±15.8 | 0.23 (-0.67 to 1.13) | 0.6 |
| Waist circumference cm | 84.6±11.7 | 84.8±11.8 | 0.51 (-0.16 to 1.19) | 0.13 |
| Body mass index kg/m^2^ | 24.1±4.8 | 24.29±4.71 | 0.17 (-0.06 to 0.4) | 0.15 |
| SBP mmHg | 108.35±12.1 | 108.86±12.9 | 0.56 (-0.54 to 1.66) | 0.3 |
| DBP mmHg | 65.4±8.1 | 65.8±8.4 | 0.33 (-0.42 to 1.09) | 0.39 |
| Fasting plasma glucose  mmol/l | 4.83±±0.37 | 4.81±0.49 | -0.02 (-0.06 to 0.01) | 0.24 |
| 2 hour post oral glucose tolerance test plasma glucose  mmol/l | 5.63±1.08 | 5.68±1.43 | 0.001 (-0.01 to 0.11) | 0.9 |
| Total cholesterol  mmol/l | 4.74±0.82 | 4.73±0.80 | -0.03 (-0.08 to 0.03) | 0.35 |
| LDL  mmol/l | 2.97±0.75 | 2.95±0.73 | -0.03(-0.08 to 0.02) | 0.2 |
| HDL mmol/l | 1.27±0.27 | 1.28±0.28 | 0.003 (-0.0.01 to 0.02 | 0.7 |
| Serum triglycerides mmol/l | 1.10±0.52 | 1.11±0.55 | 0.001 (-0.04 to 0.04) | 0.9 |

Mean difference (with SD) adjusted for baseline value, and treatment group (CI, confidence interval).

Abbreviations; SBP- systolic blood pressure DBP- diastolic blood pressure LDL-Low density lipoprotein, HDL- High density lipoprotein

Data are mean ± SD unless otherwise stated

Table S3. Percentage of participants adhering to and achieving weight loss, physical activity goals, and behavior change for physical activity and stress reduction: Pragmatic life style modification (P-LSM) as compared to control lifestyle modification (C-LSM) in 3539 healthy participants

Age 5-40 years

| Parameters | P-LSM  N=1726 | C-LSM  N=1813 | p-value |
| --- | --- | --- | --- |
| >5% Weight loss | 137 (7.6%) | 126 (6.7%) | 0.304 |
| Increased physical activity (>600 MET-min/wk) | 855 (47.3%) | 797 (42.4%) | 0.03 |
| Behavior change to increase physical activity (change in behavior from pre-action to action phase) | 603 (33.4%) | 510 (27.2%) | 0.000 |
| Behavior change for stress reduction (change in behavior from pre-action to action phase) | 267 (14.8%) | 274 (14.6%) | 0.868 |

Age <18 years

| Parameters | P-LSM  N=850 | C-LSM  N= 875 | p-value |
| --- | --- | --- | --- |
| >5% Weight loss | 53 (6.2%) | 56 (6.4%) | 0.853 |
| Increased physical activity (>600 MET-min/wk) | 338 (39.4%) | 304 (34.8%) | 0.043 |
| Behavior change to increase physical activity (change in behavior from pre-action to action phase) | 304 (35.5%) | 258 (29.5%) | 0.007 |
| Behavior change for stress reduction (change in behavior from pre-action to action phase) | 76 (8.8%) | 55 (6.3%) | 0.041 |

Age ≥18 years

| Parameters | P-LSM  N=876 | C-LSM  N=938 | p-value |
| --- | --- | --- | --- |
| >5% Weight loss | 84 (8.8%) | 70 (7.0%) | 0.127 |
| Increased physical activity (>600 MET-min/wk) | 517 (54.4%) | 493 (49.1%) | 0.020 |
| Behavior change to increase physical activity (change in behavior from pre-action to action phase) | 299 (31.4%) | 252 (25.1%) | 0.002 |
| Behavior change for stress reduction (change in behavior from pre-action to action phase) | 191 (20.1%) | 219 (21.8%) | 0.348 |
